# Gold-based Inorganic Nanohybrids for Nanomedicine Applications

**DOI:** 10.7150/thno.42284

**Published:** 2020-07-02

**Authors:** Xianguang Ding, Dan Li, Jiang Jiang

**Affiliations:** 1 Key Laboratory for Organic Electronics and Information Displays & Jiangsu Key Laboratory for Biosensors, Institute of Advanced Materials (IAM), Nanjing University of Posts and Telecommunications, Nanjing, 210023, China.; 2 Department of Dermatology, Nanjing Drum Tower Hospital, The Affiliated Hospital of Nanjing University Medical School, Nanjing, 210008, China.; 3 i-Lab and Division of Nanobiomedicine, CAS Key Laboratory of Nano-Bio Interface, CAS Center for Excellence in Nanoscience, Suzhou Institute of Nano-Tech and Nano-Bionics, Chinese Academy of Sciences, Suzhou, 215123, China.; 4 School of Nano-Tech and Nano-Bionics, University of Science and Technology of China, Hefei, 230026, China.

**Keywords:** Au nanoparticles, hybrid nanostructures, localized surface plasmon resonances, nanomedicine, theranostics

## Abstract

Noble metal Au nanoparticles have attracted extensive interests in the past decades, due to their size and morphology dependent localized surface plasmon resonances. Their unique optical property, high chemical stability, good biocompatibility, and easy functionalization make them promising candidates for a variety of biomedical applications, including bioimaging, biosensing, and cancer therapy. With the intention of enhancing their optical response in the near infrared window and endowing them with additional magnetic properties, Au nanoparticles have been integrated with other functional nanomaterials that possess complementary attributes, such as copper chalcogenides and magnetic metal oxides. The as constructed hybrid nanostructures are expected to exhibit unconventional properties compared to their separate building units, due to nanoscale interactions between materials with different physicochemical properties, thus broadening the application scope and enhancing the overall performance of the hybrid nanostructures. In this review, we summarize some recent progresses in the design and synthesis of noble metal Au-based hybrid inorganic nanostructures for nanomedicine applications, and the potential and challenges for their clinical translations.

## Introduction

Although huge economic and scientific efforts have been invested in cancer research worldwide, more than 10 million new cancer cases are diagnosed each year, with death toll continues to rise 1-4. Biomedical nanotechnology, including molecular diagnostic and nanotherapeutics that provide new options for clinical treatment, has recently been shown as a very promising technology to improve cancer patients' treatment outcomes and reduce the socioeconomic burden. Potential clinical applications of nanotechnology can be categorized depending on its usage on the body as either outside (such as “point-of-care” testing) or inside (such as diagnostics and targeted drug delivery). When focusing on their clinical application inside the body, it is highly desirable to develop strategies that enable real-time tracking of the therapeutic response during the treatment, in order to avoid insufficient treatment or over-dosing side effect. In recent years, theranostic strategies based on various nanomaterials, noble metal Au nanoparticles (NPs) in particular, have received tremendous attention 5-14. Under AC electromagnetic field (light), free charge carriers in Au NPs are driven into collective oscillations, displaying unique optical phenomena termed as localized surface plasmon resonance (LSPR). This intense light-matter interaction enables huge enhancement of local electromagnetic field, and has been widely exploited in the fields of optical imaging, sensing, and photocatalysis 15-21. Generally speaking, LSPR can be tuned over a wide spectral window from visible to the near infrared (NIR) region, depending on the NPs physicochemical properties such as size and morphology. Therefore, methods and protocols have been developed in different research labs for synthesizing Au NPs in a variety of shapes, such as nanosphere 22-24, nanocube 25, nanotriangle 26, nanocage 27, and nanoshell 28, 29. By changing their size (1 to 100 nm), shape, and structure (single particle, alloy, heterodimer, core-shell, etc.), Au nanostructures can display unique linear and nonlinear optical behaviors, enabling their use as strong photosensitizers in phototherapy and *in vitro* diagnostics 27, 30-34. Moreover, Au NPs possess large surface areas that can be conveniently functionalized with various biomolecules by means of Au-thiolate chemistry, facilitating the attachment of different moieties, such as antibodies, peptides, and biocompatible polymers with good biocompatibility and targeting capability 35, 36. The development of facile synthesis and surface functionalization strategies of Au NPs have pushed forward their practical applications in the field of nano-biomedicine, including bioimaging 37-41, drug delivery 42-44, cancer diagnosis, and therapeutics 45-50.

Hybrid nanostructures composed of multiple domains with different compositions have attracted great interests in diverse research fields. For biomedical applications, hybrid nanostructures can provide multimodal imaging modality or imaging-therapy capability all-in-one single unit. More specifically, since Au possess excellent X-ray attenuation ability and high photothermal transduction efficiency, combining Au NPs with metal oxides or metal chalcogenides would either provide complementary imaging modality for accurate cancer diagnosis or offer additional therapeutic avenue for enhanced cancer treatment, thus overcoming the limitation of single theranostic model. Hence, the combined characteristics of Au-based nanostructures would be extremely valuable for their potential applications in precision nanomedicine. Moreover, the construction of plasmonic Au NPs based hybrid nanocomposites may effectively incorporate light absorption, magnetic response, and thermal effect in one single nanostructure. The mutual interaction between Au NPs and neighboring nanomaterials at the nanoscale contact can generate complex interfacial behaviors, such as electron transfer and near-field enhancement, which may induce changes in the effective carrier concentration and optical resonances 51-54. This plasmon-driven carrier density change and near-field effect in nanohybrids can lead to potential synergistic performance enhancement when compared to the simple sum of the isolated individual components. For example, it is demonstrated that Au NPs can activate the adjacent semiconductors or metal species, enabling increased photoenergy conversion or enhanced light-absorption properties, thus promoting reactive oxygen species (ROS) generation, photoacoustic signal amplification, and heat generation 55-57, benefiting the biomedical outcomes of photodynamic therapy (PDT), photoacoustic (PA) imaging, and photothermal therapy (PTT). Therefore, designing Au-based nanohybrids is a desirable strategy to achieve enhanced theranostic efficiency without increasing the dose of NPs applied, thus averting potential side effect 58-66. These promising features together with their ease of surface modification make noble metal Au-based nanocomposites a powerful platform for diverse biomedical applications 67-73.

**Figure 1:**
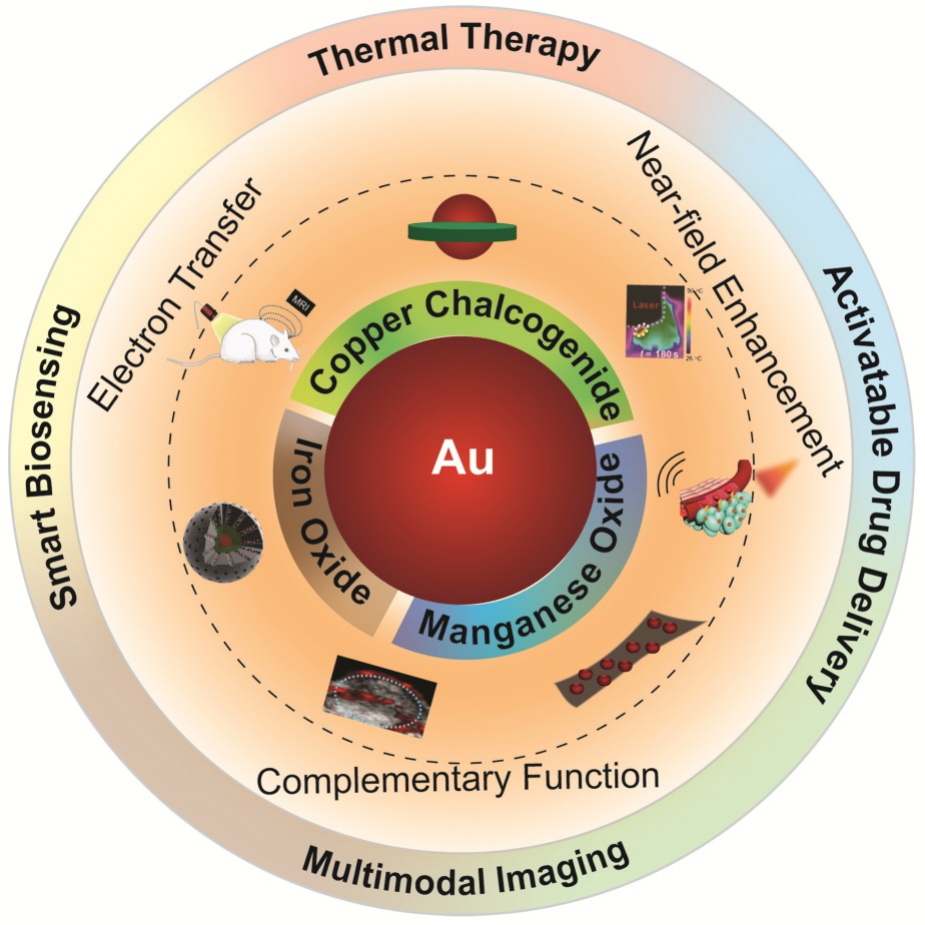
Illustration of various Au-based inorganic hybrid nanocomposites for diagnostic and therapeutic nanomedicine applications.

Some excellent reviews have summarized the advances of using noble metal NPs in the field of nanomedicine such as drug delivery, phototherapy, and biosensing 74-76. However, reviews focusing specifically on Au NPs-based inorganic hybrid nanostructures for biomedical applications are still rare. In this review, we will focus on the design and synthesis of Au-based inorganic hybrid nanostructures, and their improved performance when being applied in the field of nanomedicine, such as bioimaging, cancer therapy, and drug delivery 77-81. For the choice of adjoining components to Au, we limit our selection to copper chalcogenide, iron oxide, and manganese oxide, which are bioactive nanomaterials that can provide complementary theranostic potential to Au (as schematically illustrated in Figure 1). For each type of nanohybrid, a few important aspects will be discussed including the design and preparation of hybrid nanostructures, interaction between noble metal Au and the adjoining components, as well as their biomedical performance as theranostic agents (as briefed in Table 1).

## Au-Cu_2-x_E nanocomposites in nanomedicine

Other than the most researched noble metal nanocrystals, recent studies find that heavily-doped semiconductor nanocrystals such as non-stoichiometric copper chalcogenide NPs (Cu_2-x_E, where E = S, Se, Te; 0<x≤1) with different compositions can also support LSPR, due to their positively charged free carriers 82-86. This opens up a new field for plasmonic research 87-94, as LSPR of semiconductor nanocrystals can be easily tuned from visible to NIR by simply changing their doping levels. For biomedical applications, the emergence of copper chalcogenide nanocrystals circumvents the limitations experienced when using NIR absorbing anisotropic Au nanocrystals, which are generally large in size and unstable under laser irradiation conditions. Moreover, combining the traditional plasmonic noble metal Au with copper chalcogenide has attracted increasing attention in recent years. Many research groups have devoted efforts into constructing dual plasmonic noble metal-doped semiconductor nanocrystal hybrids, and investigated their coupled surface plasmon resonance properties and applications in the fields of catalysis and nanomedicine 77, 95, 96.

### Photothermal therapy

By changing the doping levels either chemically or electrochemically, the LSPR of Cu_2-x_E can be tuned dynamically, showing characteristic LSPR peaks extendable to the second NIR (NIR-II) window (1000-1350 nm), which is the optimal biological transparent window with larger optical penetration depth and higher maximum permissible exposure of light irradiation over the traditional NIR-I window (700-950 nm) 97-101. Through the construction of dual plasmonic nanohybrids, the LSPR coupling between Au and Cu_2-x_E may open up a new regime for designing photo-absorbers with enhanced photothermal efficiency, an attractive attribute for imaging and therapy applications in the NIR-II window.

**Figure 2:**
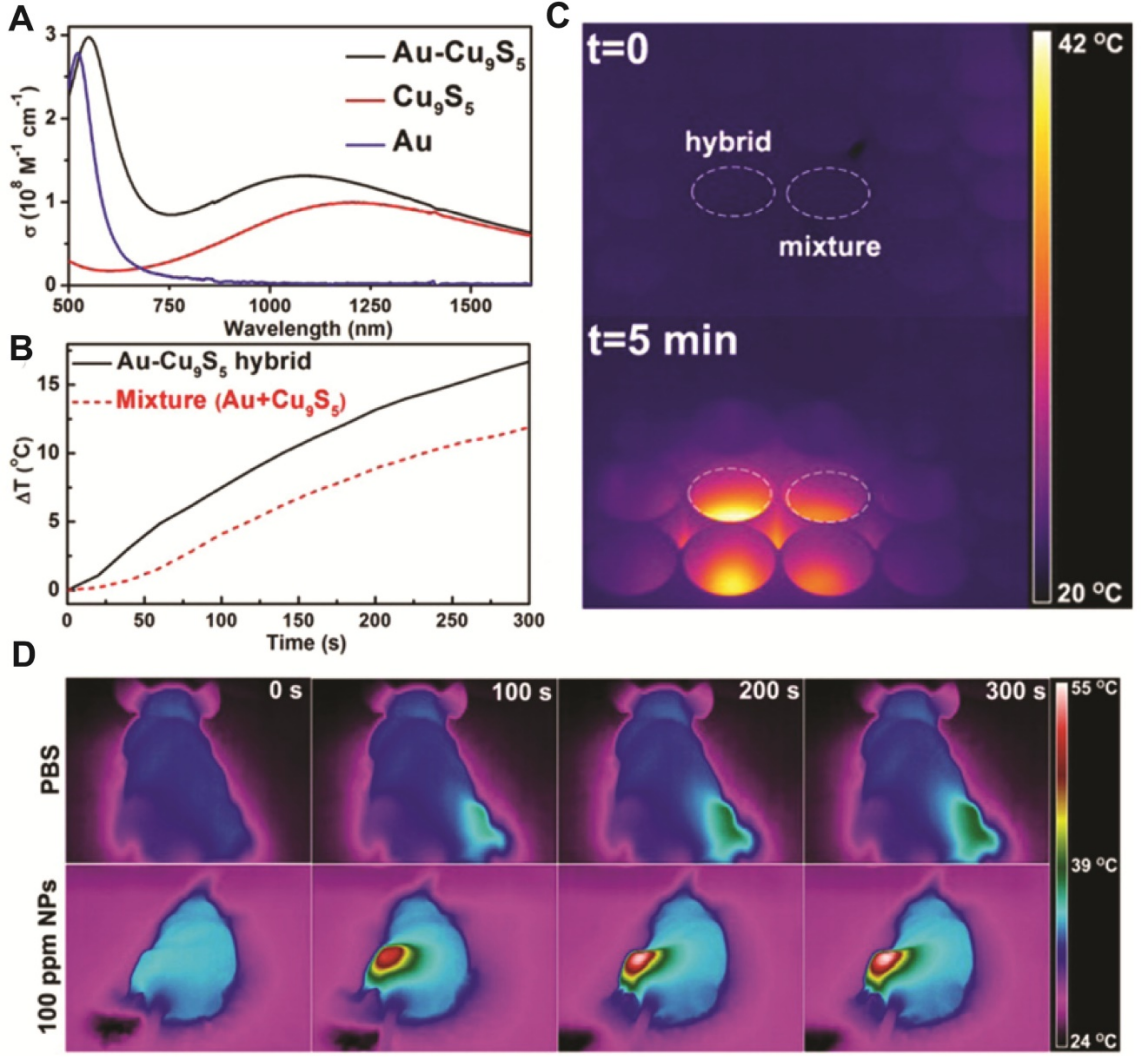
(**A**) Molar extinction coefficient of Au-Cu_9_S_5_ hybrid nanostructures and corresponding Au and Cu_9_S_5_ NPs. (**B**) Temperature increment of Au-Cu_9_S_5_ hybrid nanostructures compared to the physical mixture of Au and Cu_9_S_5_ NPs at the same concentrations. (**C**) Comparison of temperature changes captured by a thermal imaging camera from Au-Cu_9_S_5_ hybrids and the physical mixture of Au and Cu_9_S_5_ NPs under laser irradiation. (**D**) Representative thermal images of tumor-bearing mice under the irradiation of 1064 nm laser (0.6 W cm^-2^). Images are reproduced with permission from 77, copyright 2014 American Chemical Society.

In 2014, our group constructed a dual plasmonic hybrid Au-Cu_9_S_5_ with well-controlled interfaces 77. Using the high purity heterodimer nanohybrid, we investigated the LSPR coupling effect originating from the collective electron and hole oscillations in the hybrid system, and found that the synergistic interactions between two components contributed to their enhanced photothermal performance in the NIR-II window. When comparing the molar extinction coefficient of the hybrid NP to that of its individual components (Au and Cu_9_S_5_), the Au-Cu_9_S_5_ hybrid showed a 50% enhanced absorption at 1064 nm compared to pure Cu_9_S_5_ NPs synthesized using the same protocol (Figure 2A). This enhanced NIR absorption further translated to improved heating capability (Figure 2B), as shown clearly in the thermal images (Figure 2C). The light penetration depth in the NIR-II window was also explored and a decay length of 5.3 mm at 1064 nm was determined. The experimentally measured photothermal performance and theoretical calculations revealed strong LSPR interaction between Au and Cu_9_S_5_ domains in the nanohybrids. When being used for *in vivo* photothermal therapy, more than 10 °C increase was observed at tumor site under 1064 nm laser irradiation at a power density of 0.6 W cm^-2^, which is higher than the required effective temperature for cancer photothermal therapy (42-45 °C), thus inducing significant tumor ablation (Figure 2D). By combining X-ray computed tomography (CT) imaging and photothermal therapy capabilities in one nanostructure, the Au-Cu_9_S_5_ nanohybrids were demonstrated to be an attractive multifunctional platform for theranostic application. As the first report on efficient photothermal therapy in the NIR-II windows with power density lower than laser safety standards (1 W cm^-2^, ANSI Z136.1-2007, American National Standard for Safe Use of Lasers), this work reveals that constructing dual plasmonic nanostructures and optimizing the coupling effect of LSPR in nanohybrids is an efficient strategy to design better-performing theranostic agent in the NIR-II window.

Following the work of Au-Cu_9_S_5_ nanohybrids construction, various synthetic methods have been deployed to integrate Au in different shapes with copper chalcogenides to form dual plasmonic nanostructures of tunable geometries, in order to explore their LSPR coupling effect and enhanced photothermal capacity. To establish a more general strategy for synthesizing dual plasmonic nanocomposites, our group developed a facile aqueous phase synthesis route to integrate plasmonic Au with self-doped semiconductor Cu_2-x_Se 102. Using a Se-mediated approach, Au-Cu_2-x_Se hybrid nanocrystals with different Au core morphologies such as nanoparticle, nanorod, and nanotriangle can be facilely synthesized. Moreover, Au-Cu_2-x_Se hybrid nanocrystals with different morphologies such as core-shell and heterodimer geometry can be obtained by varying the polymers used for nanocrystal stabilization. Independently, Xia and coworkers developed a general and eco-friendly method to synthesize core@shell Au@Cu_2-x_E (E = S, Se) dual plasmonic nanohybrids in aqueous solution for multimodal imaging and tumor therapy applications 103. Due to the plasmonic coupling between noble metal core and semiconductor nanoshell, the as-prepared hybrid Au@Cu_2-x_S showed an extremely large extinction coefficient of 9.32 L g^-1^ cm^-1^ at 808 nm. Another approach for obtaining Au@Cu_2-x_S core@shell NPs with independently tunable core and shell morphology was developed by Zhang *et al*., through a cation exchange enabled non-epitaxial strategy 95, where the nonstoichiometric composition and thickness of the Cu_2-x_S shell can be precisely controlled.

### Photoacoustic imaging

Photoacoustic (PA) imaging modality is based on measuring the acoustic waves generated in biological tissues after short laser pulses excitation 104-106. By combining the advantage of high spatial resolution from optical imaging and large penetration depth from ultrasound detection, PA imaging has become a fast-developing imaging technique with great potential in biomedical and clinical applications 41. PA imaging contrast depends on the optical cross sections of the tissue and the injected imaging agents. Therefore, strongly absorbing plasmonic nanocrystals including Au and copper chalcogenides have been selected as candidates for PA imaging contrast enhancing agents 107-109.

**Figure 3:**
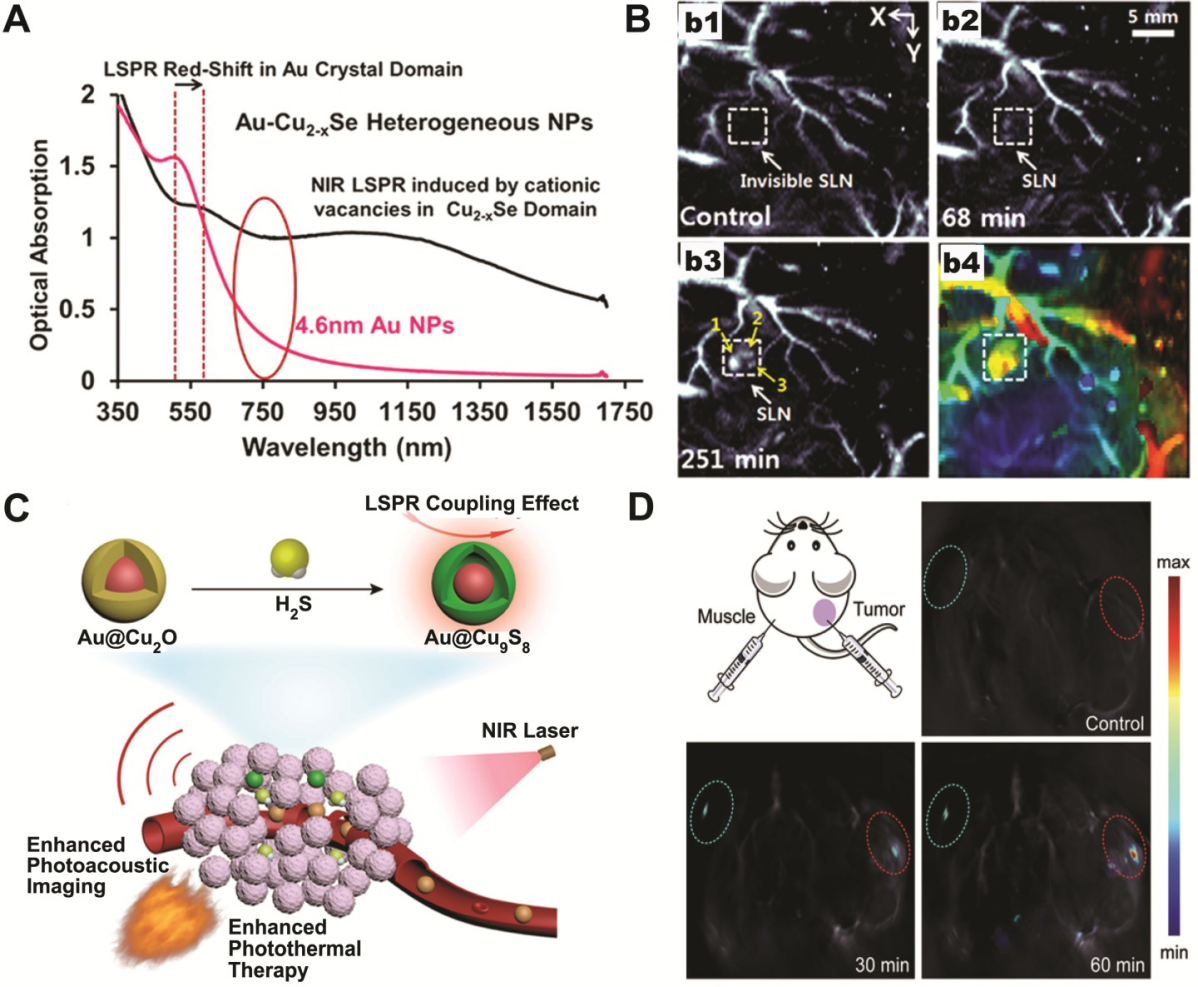
(**A**) UV-vis spectra of Au-Cu_2-x_Se heterodimers and the 4.6 nm Au seed NPs. (**B**) Representative PA imaging of sentinel lymph node before (b1) and after Au-Cu_2-x_Se injection for 68 min (b2) and 251 min (b3), and the depth-encoded PA coronal image (b4). Images are reproduced with permission from 78, copyright 2013 American Chemical Society. (**C**) Schematic illustration of endogenous H_2_S-triggered enhanced PA imaging and photothermal therapy based on LSPR coupling effect. (**D**) *In situ* sulfidation of Au@Cu_2_O nanocomposites and the corresponding PA images before and after intratumoral injection of Au@Cu_2_O nanostructures at different time points. Images are reproduced with permission from 81, copyright 2019 John Wiley and Sons.

Based on the plasmonic coupling induced enhanced photothermal response of Au-copper chalcogenides nanohybrids, Swihart and coworkers have reported using Au-Cu_2-x_Se heterodimer nanocrystals as contrast agents for deep tissue PA imaging 78. The Au-Cu_2-x_Se heterodimer NPs exhibited a broad optical absorption spectrum across both NIR-I and NIR-II window, as a result of electron transfer between the constituting Au and Cu_2-x_Se domains (Figure 3A). Under 1064 nm excitation, with a power density (10 mJ cm^-2^) at only 1/10 of the ANSI safe limit, sentinel lymph node (SLN) mapping up to 17 mm under skin was achieved (Figure 3B), demonstrating their potential for clinical applications.

As photothermal and photoacoustic effects are intrinsically related to the light-matter interaction, the dual plasmonic Au-copper chalcogenides nanohybrids are perfect candidates for PA imaging-guided photothermal therapeutic applications operating at the same NIR window. Nie *et al.* reported aqueous phase synthesis of Au@Cu_2-x_S core-shell NPs via anion exchange between S^2-^ and Au@Cu_2_O core-shell NPs, which were then used for accurate tumor identification and efficient ablation through PA imaging-guided photothermal therapy (Figure 3C) 73. The idea of chemical conversion from Cu_2_O to CuS using S^2-^ was further utilized for smart theranostic agent design 81. A characteristic physiological feature of colon cancer is the high level of endogenous hydrogen sulfide (H_2_S). Yang and coworkers have shown that the photothermal conversion efficiency of Au@Cu_2_O increased 50% in the presence of NaHS. Moreover, after intratumoral or intravenous injection, *in situ* sulfidation of Au@Cu_2_O by endogenous H_2_S in colon tumor was confirmed by both photoacoustic imaging (Figure 3D) and Raman spectroscopy. The converted Au@Cu_9_S_8_ showed about twice stronger absorption at 808 nm, with increased photothermal conversion efficiency ~1.2 times higher than the original Au@Cu_2_O. This work demonstrates that the *in situ* generated Au-copper chalcogenides nanohybrids, formed by responding to local physiological niche environment at tumor site, can act as smart PA imaging-guided photothermal theranostic agent to treat cancers.

### Activatable drug delivery

Photothermal therapy can be used to eradicate tumor cells through localized heating. However, unsatisfactory tumor inhibition may occur due to inhomogeneous heating effect at tumor site. Combining chemotherapy with photothermal therapy has shown great promise in cancer treatment, where local heating can be used to regulate drug release with both spatial and dosage control, while the elevated local temperature also improves drug efficacy in treating cancer. To enable higher drug loading capacity, voids are often introduced into the nanohybrid design. For instance, Lin *et al*. described the synthesis of hollow CuS@Cu_2_S@Au nanostructures, which not only exhibited enhanced photothermal conversion efficiency, but also afforded high drug loading capability by providing large cavity and mesoporous shell, thus enabling photo-responsive drug release under NIR laser excitation 96. Zhang's group developed Au@void@CuS yolk-shell nanostructures as multifunctional drug carriers 110. After doxorubicin (DOX) loading, the resultant DOX-Au-CuS yolk-shell nanocomposites could kill cancer cells more efficiently than the unloaded NPs under the same 980 nm laser irradiation conditions, due to the simultaneous photothermal and chemotherapeutic effect.

Drug delivery vehicles are often responding only to single stimulus such as external light irradiation or endogenous pH environment. Designing drug delivery system responding to multiple stimuli will not only minimize the undesirable release of chemotherapeutics thus avoiding adverse side effects, but also maximize the drug dosage in the target region with improved drug availability. To reach this goal, Cao *et al*. designed a dual responsive drug release system utilizing a rattle-type Au@Cu_2-x_S hollow mesoporous structure 111. With this structure, a high drug loading capacity of 908 μg DOX per mg of the hollow mesoporous nanocrystals was achieved. More importantly, the obtained hybrid nanostructures displayed both endogenous pH- and external photo-responsive drug release behaviors. The acidic pH mimicking tumor microenvironment and NIR laser irradiation could activate the drug delivery system with over 70% of DOX release in 20 min. This makes Au@Cu_2-x_S hollow mesoporous structure a promising agent for chemo-photothermal therapy under photoacoustic imaging guidance, due to their superb photothermal conversion efficiency and conspicuous capability of photo activatable drug release property.

**Figure 4:**
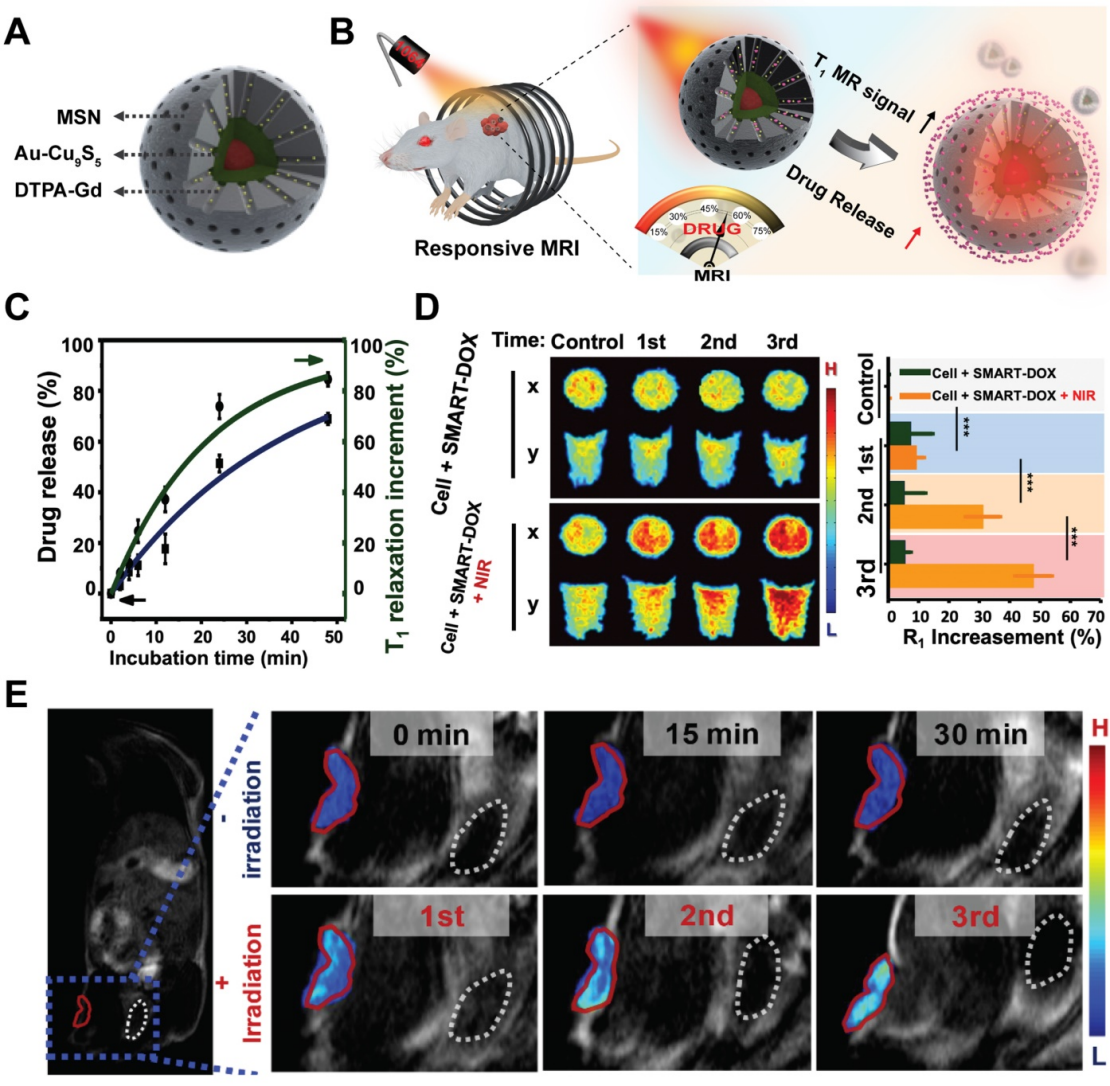
Schematic illustration of Au-Cu_9_S_5_@MSN nanostructures (**A**) and their NIR responsive drug release behavior with real time MRI monitoring property (**B**). (**C**) DOX release from Au-Cu_9_S_5_@MSN-DOX nanocomposites at 45 °C, and the corresponding T_1_ relaxation increment. (**D**) Evolving T_1_-weighted MR images and MR relaxations of cancer cells treated with Au-Cu_9_S_5_-DOX nanocomposites after different repetition of NIR irradiations. (**E**) T_1_-weighted MR images of mice injected with Au-Cu_9_S_5_-DOX nanocomposites with and without laser irradiation treatments. Images are reproduced with permission from 80, copyright 2019 Springer Nature.

Further integrating activatable drug delivery system with real-time drug release monitoring function would enable patient-specific drug administration and benefit personalized medical treatment. Recently, based on the Au-Cu_9_S_5_ nanostructures, we have developed a smart drug delivery platform with noninvasive activatable magnetic resonance (MR) imaging capacity for controllable drug release tracking 80. The smart nanocomposites contained two functional components, which were the inner Au-Cu_9_S_5_ core for heat generation under laser irradiation and outer mesoporous silica (MSN) shell for drug molecules loading and paramagnetic Gd^3+^ ions anchoring (Figure 4A). The paramagnetic Gd^3+^ ions-based chelates were used clinically to accelerate the longitudinal relaxation (T_1_) of excited protons, thus increasing the longitudinal relaxation rate (r_1_) and generating enhanced T_1_ MR images. Under exogenous NIR-II irradiation, localized heating of Au-Cu_9_S_5_ core would melt the gatekeeper phase-change materials loaded in the MSN shell and trigger pulsated drug release with good on/off control (Figure 4B). With the released drug molecules leaving the hybrid nanocomposites, the accessibility of proton to the paramagnetic Gd^3+^ ions anchored in the mesoporous channels was significantly promoted, which improved r_1_ of protons, and resulting in a positive correlation between T_1_ MR imaging signal and the amount of released drugs (Figure 4C), which was further verified at cellular (Figure 4D) and *in vivo* levels (Figure 4E).

## Au-Fe_x_O nanocomposites in nanomedicine

Iron oxide (Fe_2_O_3_ and Fe_3_O_4_) with good chemical stability and biocompatibility has gained tremendous attention in diverse biomedical applications including magnetic resonance imaging, sensing, remote-controlled drug delivery, and magnetic hyperthermia 112-119. By integrating magnetic iron oxide and plasmonic Au into one single unit, the as formed magneto-plasmonic hybrid nanostructures possess great potential in theranostic applications. Their optical and magnetic properties can be tuned independently by changing the respective nanoparticle domain size, shape, composition, and geometry. The unique attributes of magneto-plasmonic hybrid nanostructures have attracted great interests into their design and application for simultaneous diagnosis and treatment of cancer.

### Thermal therapy

Thermal therapy is a promising approach to kill cancer cell with the local temperature at tumor site reaching 42-45 °C. Iron oxide and Au NPs are typical thermal agents that can generate heat to destroy tumor cells through noninvasive interaction with either oscillating magnetic field or NIR light 120. However, both magnetic hyperthermia and photothermia have their inherent drawbacks. While noble metal Au-based photothermia show high heating efficiency with good spatial resolution, the compromised light penetration depth in living tissues set a limit on its potential clinical applications. On the other hand, magnetic NP mediated hyperthermia employs radiofrequency, thus overcoming the penetration depth limitation of photothermia. Unfortunately, magnetic hyperthermia utilizing biocompatible iron oxide NPs suffers from their low specific loss powers. Extensive efforts have been put into modulating the size, magnetization or anisotropy of magnetic particles to enhance their specific absorption rate, thereby improving their heat generation capacity. Designing magneto-plasmonic nanostructures through hybridizing noble metal Au with magnetic nanomaterials together has been explored to overcome the limitations set by the individual components. It was found that by capping magnetic NPs with Au, more local heat could be generated when the hybrids were put under a radiofrequency field. Challa S. S. R. Kumar *et al*. demonstrated that superparamagnetic iron oxide (Fe_3_O_4_) NPs (SPION, 5.4 nm) coated with 0.4-0.5 nm thick gold nanoshell can generate 4-5 times more heat compared to that of the pure Fe_3_O_4_ NPs under a low-frequency oscillating magnetic field 121. They speculated that the higher heat generation capacity was attributed to larger magnetic anisotropy of the superparamagnetic Fe_3_O_4_ NPs inside Au shell. Zhou's group reported a similar study recently 122. Under the same magnetic induction conditions, a local temperature of 15 °C higher was achieved with the hybrid Fe_3_O_4_/Au cluster/shell nanostructures compared to that of the pure Fe_3_O_4_ NPs, and induced higher percentage of cancer cell apoptosis. Furthermore, the Fe_3_O_4_/Au nanostructures possessed high transverse relaxation rate (r_2_) for MR imaging (MRI), while the Au nanoshells can be used as surface enhanced Raman scattering (SERS) substrate for early diagnosis. SERS is a surface enhanced optical phenomenon, as Raman signals from surface-absorbed molecules are significantly amplified, due to the strongly enhanced near-field on the surface of noble metal NPs as the result of LSPR excitation. SERS allows optical sensing with high spatial resolution and sensitivity down to single molecule level under optimal conditions. Other than improving magnetic hyperthermia, photothermal effect can also be magnetically amplified via magnetophoretic manipulation strategy, as illustrate in the work by Sepúlveda *et al*. 123, where the optical heating efficiency of the Fe/Au nanodomes could be dramatically enhanced by local NPs enrichment in the laser irradiation zone under the assistance of an external magnetic field. In addition to single hyperthermia modality, magnetic hyperthermia and photothermia can be synergistically integrated in a properly designed magneto-plasmonic nanohybrid. Abou-Hassan and coworkers synthesized Au nanoshell coated iron oxide multi-core magneto-plasmonic nanohybrids with diameter of around 30 nm 124. They demonstrated that the heat generated by the magneto-plasmonic nanohybrids display a cumulative effect when both magnetic and plasmonic heating modalities are working simultaneously. With the treatment dose only 1/10 of that used in typical magnetic hyperthermia therapy, a rapid temperature increase to 48 °C could be achieved in tumor tissue under simultaneous magnetic induction and laser irradiation treatment.

### Multimodal MR/(CT, PA, SERS) Imaging

Biomedical imaging is important for early diagnosis and treatment evaluation, which has emerged as a key technology for the development of targeted therapies. Combining multiple imaging tools together can be very helpful in personal and precision medicine. While molecular imaging tools such as positron emission tomography (PET), computed tomography (CT), and FO (fluorescence optical) imaging have been widely used in clinical diagnostics, each of these imaging methods possesses its own strengths and weaknesses. In recent years, integrating different imaging modalities together by designing multimodal imaging agents such as CT/MR, FO/MR, PA/MR, and SERS/MR have been suggested to obtain more comprehensive pictures for accurate cancer diagnosis 125-132. By integrating magnetic nanomaterials with Au, the concomitant MR imaging modality can provide non-invasive imaging, large penetration depth, and good soft tissue contrast.

Hybrid Au-Fe_x_O nanocomposites are considered potential bimodal CT/MR imaging agents 133-135, where Fe_x_O component serves as T_1_ or T_2_ MR contrast enhancer, while Au with efficient X-ray attenuation capability works as powerful CT contrast enhancing agent. Gu *et al*. fabricated Au-Fe_3_O_4_ heterostructures for bimodal MR/CT imaging application by a seeded-growth method 136. The prepared Au-Fe_3_O_4_ heterostructures were composed of 14 nm Fe_3_O_4_ attached to 11 nm spherical Au NPs. The r_2_ value of the heterostructures was determined to be 136.4 mM^-1^ s^-1^ at 1.5 T. Using a rabbit model, the Au-Fe_3_O_4_ heterostructure composites exhibited excellent MR/CT contrast enhancing performance. The rabbit liver can be clearly observed by MR imaging. Meanwhile, the detailed anatomical structures such as rabbit right ventricle can be clearly viewed by CT imaging. Using a facile one-pot strategy, Shi *et al*. reported core-shell Fe_3_O_4_@Au nanostructures for bimodal MR/CT imaging application 137. The MR and CT performance evaluation showed that the hybrid NPs possess high r_2_ relaxivity (146.07 mM^-1^ s^-1^) and excellent X-ray attenuation ability, which was then successfully applied to aorta CT imaging and liver MR imaging in mouse models. In another study, the same group fabricated Fe_3_O_4_/Au nanocomposites based on a layer-by-layer (LBL) strategy 135. Their results demonstrated that at the optimized molar ratio of Au to Fe_3_O_4_, Fe_3_O_4_/Au NPs exhibited excellent X-ray attenuation characteristics and a relatively high r_2_ relaxation rate of 92.67 mM^-1^ s^-1^. By further modifying them with targeting molecule folic acid, the hybrid nanocomposites could be specifically uptaken by cancer cells that over express folic acid receptors on cell membrane surface. Similarly, Zhang and Wang's group reported the use of lectin conjugated Fe_2_O_3_@Au as bimodal MR/CT imaging agent *in vivo*, targeting specifically the colorectal cancer 138.

Besides attenuating X-rays for CT imaging, Au is also excellent PA imaging agent due to their LSPR characteristics. The construction of bimodal MR and PA molecular imaging agents can overcome the limitation of finite penetration depth of PA imaging, and provide structural and functional information of disease with high resolution and sensitivity. Melancon *et al*. fabricated multifunctional superparamagnetic Fe_3_O_4_@Au nanoshells with excellent PA imaging performance and high r_2_ relaxivity of 208 mM^-1^ s^-1^ 139. Based on the high NIR absorption and strong magnetic properties of Fe_3_O_4_@Au nanoshells, the hybrid Fe_3_O_4_@Au nanoshells were capable of lighting up tumor region with PA-MR imaging. Moreover, the bimodal PA and MR imaging can be used to monitor the therapeutic treatment outcome mediated by the photothermal effect of Fe_3_O_4_@Au. Functionalizing Au-Fe_x_O nanocomposites with targeting ligand could further improve their diagnostic capability. Franchini *et al*. synthesized a multilayered Fe_3_O_4_@SiO_2_@Au core-shell nanostructure conjugated with folic acid 140. With hydrodynamic diameter of 222±1.5 nm, the as-prepared nanostructure showed bimodal MR/PA imaging ability. After systemic injection into a tumor bearing mice, PA imaging revealed that Fe_3_O_4_@SiO_2_@Au had exclusively accumulated in the ovarian cancer region after 4 h. These studies demonstrate the great potential of utilizing Au-Fe_x_O hybrid nanostructures as bimodal MR/CT(PA) imaging agent for *in vivo* diagnostic applications. It is noteworthy that the biomedical imaging performance of hybrid nanostructures is strongly associated with their specific geometric arrangement. While both Au-Fe_3_O_4_ heterostructure and Fe_3_O_4_@Au core-shell nanostructure can be used for bimodal MR/PA imaging, the core-shell Fe_3_O_4_@Au nanostructures with LSPR located in the NIR region are obviously more suitable for biomedical MR/PA imaging.

**Figure 5:**
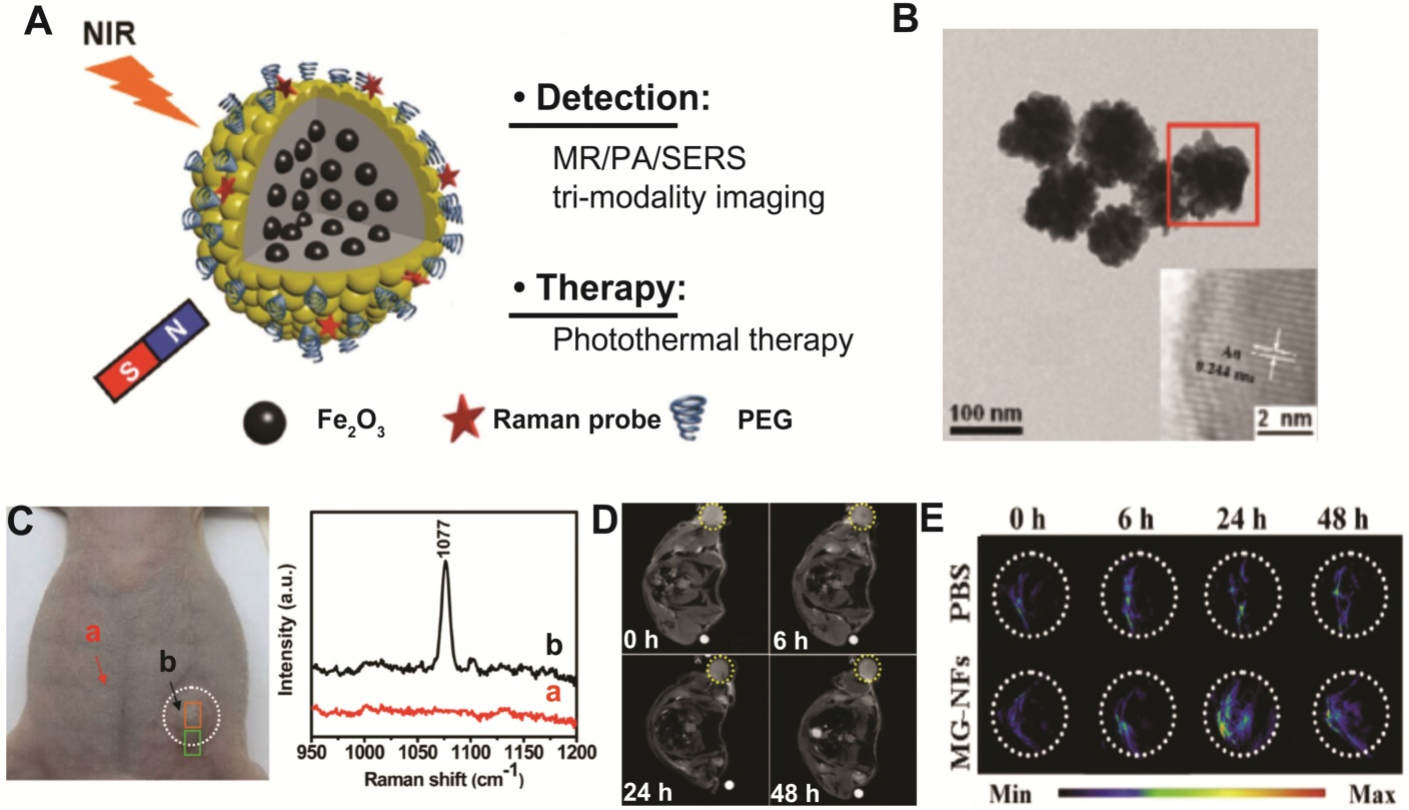
(**A**) Schematic illustration of Fe_2_O_3_@Au core-shell nanoflowers for multimodal imaging-guided tumor therapy. (**B**) Typical TEM image of Fe_2_O_3_@Au nanoflower structures. *In vivo* SERS spectra (**C**) from normal tissue (a) and tumor region (b), T_2_-weighted MR images (**D**), and PA images (**E**) of a 4T1 tumor bearing mouse injected with either Fe_2_O_3_@Au nanoflowers or PBS. Images are reproduced with permission from 141, copyright 2015 John Wiley and Sons.

Zhang's group has investigated the potential of using Au-Fe_x_O hybrid nanocomposites as multimodal SERS/PA/MR imaging-guided photothermal therapeutics by designing Fe_2_O_3_@Au core-shell structure (Figure 5A-B) 141. The combined tri-modal imaging modality (SERS/PA/MR) can provide complementary information of anatomical tumor localization and tumor resection margin for accurate tumor diagnosis and surgical treatment guidance (Figure 5C-E). Furthermore, due to strong NIR absorbance derived from Au nanoshell, Fe_2_O_3_@Au core-shell nanostructures show high photothermal transduction efficiency for cancer therapy. The 4T1 tumor bearing mice administered with Fe_2_O_3_@Au core-shell nanostructures have shown significant photothermal tumor ablation under 808 nm laser irradiation. These results illustrated that rationally designed magneto-plasmonic hybrid nanostructures can be used for efficient multimodal imaging-guided photothermal cancer therapy.

### Activatable drug delivery

**Figure 6:**
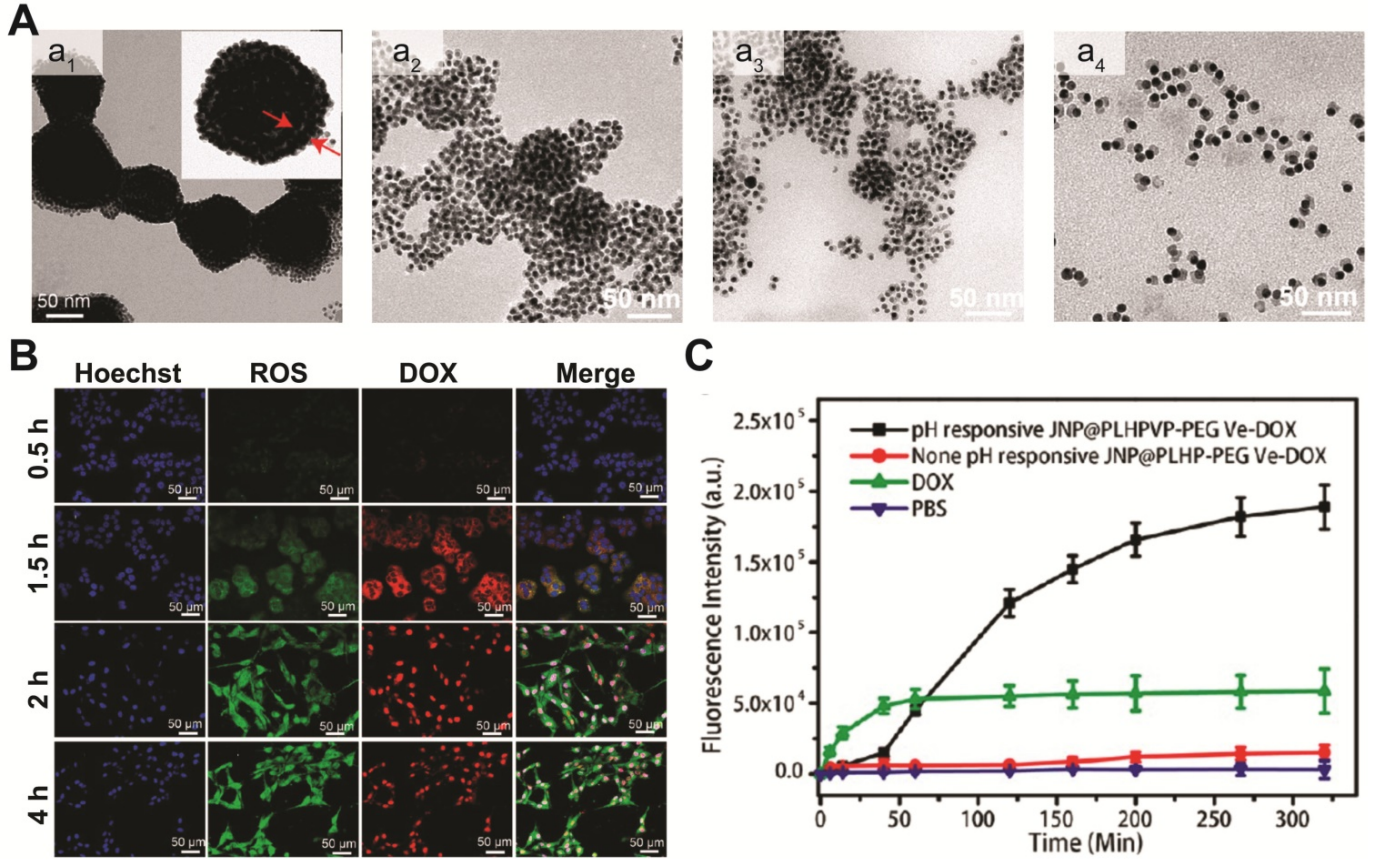
(**A**) Representative TEM images of assembled Fe_3_O_4_-Au janus structures (a_1_) after being incubated in acidic solution (pH=5.4) for 30 (a_2_), 60 (a_3_), and 90 min (a_4_). (**B**) Representative fluorescence images of tumor cells incubated with Fe_3_O_4_-Au-DOX nanocomposites at different time intervals. (**C**) Quantification of released DOX by measuring its fluorescence signals. Images are reproduced with permission from 79, copyright 2019 American Chemical Society.

Under external stimulus such as NIR light and magnetic field, the multifunctional Au-Fe_x_O hybrid nanostructures can not only act as *in vivo* diagnostic imaging agent, but also serve as powerful delivery vehicles for controlled drug release. In recent years, efforts have been put into developing different Au-Fe_x_O hybrid nanostructures with high drug loading capacity, versatile targeting ability, and smart drug release capability. Chen and coworkers reported the synthesis of a yolk-shell plasmonic-magnetic hybrid theranostic platform 142, which was composed of a small Fe_3_O_4_ core encapsulated inside a hollow cavity formed by a porous Au nanoshell. With a relative small size of around 65 nm, the yolk-shell Fe_3_O_4_-Au NPs displayed a high r_2_ value of 149.4 mM^-1^ s^-1^, which is ~2.4 times of that from the core-shell structures, indicating that the interfacial interaction of the two components can greatly affect their magnetic properties. In addition, the hollow cavity can be an ideal storehouse for drug loading. After constructing a gatekeeper on the surface using thermosensitive poly(N-isopropylacrylamide-*co*-acrylamide), thermal responsive drug release is achieved under NIR light irradiation. Initially, only weak DOX fluorescence was observed in the cells as a result of fluorescence quenching by Fe_3_O_4_-Au. Upon NIR exposure for 5 min, both cytoplasm and nucleus of the cells displayed strong red fluorescence, suggesting DOX was released, which was also confirmed in the *in vivo* study. In addition to light trigged drug delivery, pH responsive release system has also been widely adopted in different drug carrier designs. Recently, the same group developed a magnetic-plasmonic bilayer vesicle by assembling Fe_3_O_4_-Au janus structure with a pH-responsive polymer for multimodal imaging-guided cancer therapy 79. The large hollow cavity formed in the assembled bilayer structures enables a high DOX loading capacity. Due to inter-particle plasmonic and magnetic coupling, the assembled bilayer structures displayed enhanced light absorption and high T_2_ relaxivity, and exhibited improved MRI/PA contrast and photothermal activity, compared to the individual components. Moreover, the bilayer vesicles can be disassembled in mildly acidic microenvironment (Figure 6A). Therefore, DOX loaded in the hollow cavity can be released from the Fe_3_O_4_-Au-DOX bilayer vesicles, in response to the decreased pH level in tumor microenvironment (Figure 6B-C). Other than loading chemotherapy drugs, Au-Fe_3_O_4_ hybrid nanostructures can also carry singlet oxygen (^1^O_2_) photosensitizers, and act as a potential agent for photodynamic therapy. For example, Rosa-Pardo *et al*. designed a core-shell Fe_3_O_4_@Au@mSiO_2_ nanostructure with photosensitizer Rose Bengal (RB) encapsulated inside mesoporous silica 143. Due to the surface plasmon sensitization effect of Au shell, a 1.5-fold enhanced ^1^O_2_ generation by RB was detected. Furthermore, Au-Fe_3_O_4_ nanocomposites are also efficient ROS generating agents with their intrinsic enzyme-mimic characteristics 144-146. By deliberately designing hybrid nanostructures with multi-enzymatic activities to achieve cascade reactions, high chemo-dynamic therapeutic efficiency has been demonstrated using inorganic nanohybrids. For example, Shi and Chen *et al*. constructed mesoporous silica coated Au-Fe_3_O_4_ nanostructures 145. In tumor microenvironment, the Au domain behaves as glucose oxidase-mimicking nanozyme, catalyzing glucose to H_2_O_2_ and gluconic acid. At the same time, the adjacent Fe_3_O_4_ domain acts as peroxidase-mimicking nanozyme, reacting with the *in situ* generated H_2_O_2_ and producing highly toxic ROS to kill cancer cells.

## Au-MnO_2_ nanocomposites in nanomedicine

Besides iron oxides family, manganese oxides such as MnO, MnO_2_, and Mn_3_O_4_ have also been considered as promising candidates for biomedical applications 147-152. As Mn is one of the essential trace elements in human body, Mn-based nanoparticles such as MnO_2_ can be utilized and metabolized by the human body. Mn-based complexes are considered as very promising clinical agents for T_1_ MR imaging. For instance, Mn-dipyridoxyl diphosphate (DPDP) complex Mangafodipir has already been approved as an efficient T_1_ MR agent for liver imaging. In addition, MnO_2_ nanomaterials can respond to tumor microenvironment cues such as hypoxia, acidosis, and vascular endothelial growth factor, which can be utilized to amplify their diagnostic and therapeutic performance. For example, MnO_2_ nanosheets will rapidly decompose and release Mn^2+^ under mildly acidic and reducing conditions, thus enhancing the contrast of T_1_ MR imaging 153, 153. Moreover, the released Mn^2+^ could initiate Fenton chemistry to kill cancer cells by catalyzing tumor endogenous H_2_O_2_ into toxic reactive oxygen species 149. Therefore, combining MnO_2_ nanomaterials with the unique LSPR characteristics of noble metal Au can provide a promising theranostic platform as well as form smart probes for versatile biomedical applications both *in vitro* and *in vivo*.

### Enhanced radiotherapy/photodynamic therapy

Au-MnO_2_, a new kind of smart therapeutic agent, may serve as a potential theranostic candidate in the field of nanomedicine based on their good biocompatibility and tumor microenvironment responsive behaviors 149, 153-157. The heavy atom Au can absorb X-rays to generate charged particles, and enhance the effect of radiotherapy (RT). Meanwhile, the MnO_2_ domain can react with endogenous H_2_O_2_ in tumor microenvironment to generate oxygen locally, thus overcoming hypoxia-associated RT resistance. For this purpose, Liu *et al*. designed Au@MnO_2_ nanostructures 158, and they observed that Au@MnO_2_ hybrids indeed have enhanced radiotherapy efficiency as designed. In addition, the nanocomposites containing Au and MnO_2_ NPs also showed enhanced performance as photodynamic agents 159. In treating metastatic triple-negative breast cancer, core-shell Au nanocage@MnO_2_ structures were able to boost immunogenic photodynamic therapy (PDT), thus inhibiting tumor growth and metastases. The enhanced therapeutic efficiency is attributed to the tumor microenvironment responsive oxygen generating MnO_2_ components, which was decomposed at acidic tumor H_2_O_2_-rich conditions and produced sufficient oxygen to boost PDT effect originating from the adjoining Au nanocage.

### Responsive imaging

Novel tumor microenvironment-responsive imaging agents have emerged as a promising class of theranostic agent for imaging-guided cancer treatment. By designing tumor microenvironment responsive nanoprobes, large off/on imaging contrast can be achieved. For example, Meng *et al*. prepared Au nanostar@MnO_2_ nanosheet hybrid structure of less than 50 nm in dimension 160. With strong light absorption from 300 to 800 nm, the as synthesized hybrid can destroy tumor cells effectively through photothermal effect. Furthermore, the hybrid nanostructures displayed enhanced MR imaging capability in the presence of GSH, due to their redox environment responsive MR imaging capability. This study demonstrates the potential of Au-MnO_2_ nanocomposites as efficient theranostic nanoprobes for activatable MR imaging-guided photothermal therapy. Other than photothermal therapy, many other therapeutic modalities such as photodynamic therapy and chemotherapy have also been integrated with MnO_2_ components. Lin's group designed MnO_2_-Pt@Au_25_ nanocomposites, which combined photodynamic therapy, chemotherapy, and activatable MR imaging together in one system 161. The MnO_2_ nanosheets acted as carrier for both photosensitizer Au_25_ and prodrug Pt(IV) loading. In the reducing tumor microenvironment, high level of GSH would be consumed through redox reaction with MnO_2_ nanosheets and Pt(IV) prodrugs. As a result, both photodynamic therapeutic efficiency induced by Au_25_ cluster and Pt(II) chemotherapy efficiency were enhanced. More importantly, the reduced Mn(II) ions released from MnO_2_ nanosheets can increase the MR relaxivity from 401.9 mg^-1^ s^-1^ (r_1_) and 48.8 mg^-1^ s^-1^ (r_2_) to 471.3 mg^-1^ s^-1^ (r_1_) and 49.6 mg^-1^ s^-1^ (r_2_), thus enhancing their corresponding T_1_ and T_2_ MR imaging contrasts. Our group has also reported a feasible strategy to decorate various core materials including Au nanoparticle and Au nanorod with the tumor microenvironment-responsive MnO_2_ shell, which can be utilized as activatable MRI-PTT theranostic platforms for cancer therapy 162.

### Smart biosensing platform

**Figure 7:**
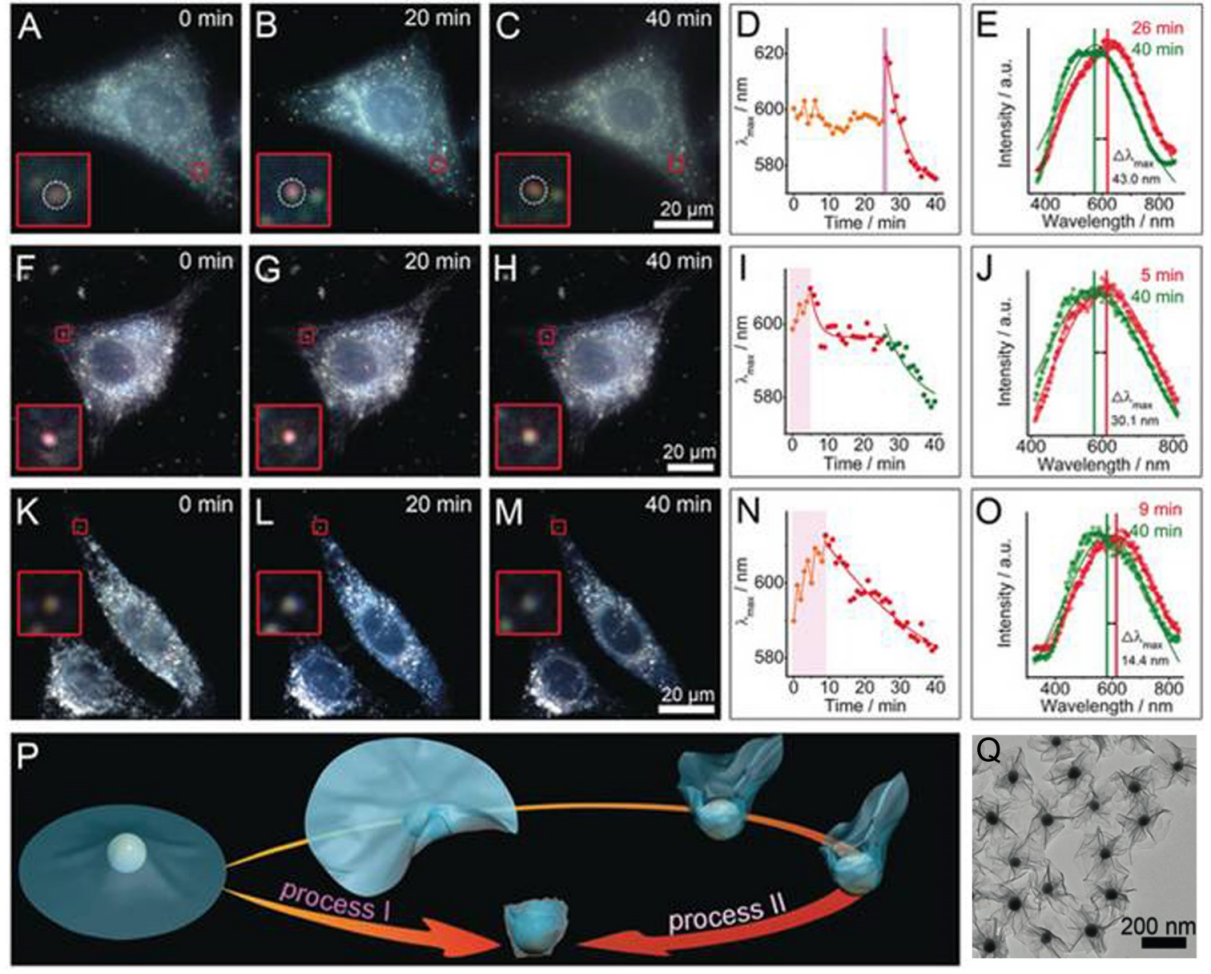
Interactions of the UFO-shaped 2D Au-MnO_2_ nanostructures with different living cells probed by dark-field images and scattering spectra: (**A-E**) HepG2 cells, (**F-J**) 3T3 cells, and (**K-O**) buthionine sulfoximine (a GSH inhibitor) pre-treated HepG2 cells. (D, I, N) Time-dependent λ_max_ of the scattering spectra changes (E, J, O) after entering the cells. (**P**) Schematic for two different types of transmembrane processes. (**Q**) TEM image of UFO-shaped 2D Au-MnO_2_ nanostructures. Images reproduced with permission from 163, copyright 2019 John Wiley and Sons.

Under a dark-field microscope, a special condenser is used to block central light so that a circular light cone is incident on the object at high angle, only allowing oblique rays to hit the object. This blocks zeroth order light, and objects scatter light more strongly will stand out from the non-scattering dark background. Therefore, Au NPs with strong light-matter interaction due to their LSPR characteristics are perfect objects to be imaged under a dark-field microscope. In this regard, Au-MnO_2_ nanohybrids are gaining interest as smart biosensors for probing complex cellular events. Xia and coworkers developed UFO-shaped Au-MnO_2_ plasmonic supraparticles with diameter of around 230 nm, and used these anisotropic structures as dark-field contrast agents to probe the nano-bio interaction at the single cell level (Figure 7A-O) 163. Due to the flexibility of thin MnO_2_ nanosheets, they can be physically deformed and folded during the endocytosis process. By employing dark-field spectroscopy, they visualized the interactions between 2D Au-MnO_2_ nanostructures and living cells, and identified two definitely different trans-membrane processes (Figure 7P). During the cell membrane wrapping process, the deformation and folding of the thin MnO_2_ nanosheets (Figure 7Q) induced effective refractive index changes around Au NPs, rendering the NPs LSPR scattering red shift with different magnitudes depending on the endocytosis process. On the other hand, the presence of redox species within cells would disintegrate MnO_2_, and induce a LSPR blue-shift, which could be employed to mark the complete cell membrane engulfment process. This LSPR modulation approach provides a convenient but efficient way to monitor the dynamic interactions between nanomaterials and cells. In addition to serve as a cellular probe, smart Au-MnO_2_ nanocomposites can also be employed for point-of-care testing. Au@MnO_2_ hybrid nanocomposites have been developed to detect ascorbic acid (AA) in human serum 164. The redox reaction between Au@MnO_2_ nanocomposites and AA resulted in the degradation of MnO_2_, inducing both MR signal increase and fluorescence recovery due to free Mn^2+^ ions released from Au clusters. This Au-MnO_2_ nanocomposite-based magnetic/fluorometric bimodal biosensor allows detection of AA in human serum with cross-validation.

## Other Au NPs-based nanocomposites

In addition to the above mentioned hybrid nanostructures, some other Au-based nanocomposites including Au-ZnO, Au-TiO_2_, and Au-reduced graphene oxide (rGO) have also been developed and utilized as new type of theranostic platforms for biomedical application 165-169. Metal oxide NPs such as ZnO and TiO_2_ can absorb photons and create electron-hole pairs, generating ROS to inhibit microbial or cancer growth. However, they only absorb in the UV region, and their capability of ROS generation is limited by fast electron-hole recombination. By integrating ZnO or TiO_2_ with Au NPs, their optical absorption can be enhanced due to LSPR effect, the spectral window is extended to the visible, and the photo generated charge carrier recombination is greatly suppressed, leading to enhanced photocatalytic and PDT activity. For example, Yin *et al.* synthesized ZnO/Au hybrid nanostructures using a photo-reduction method 165. It was found that even Au NPs of sizes less than 3 nm deposited on ZnO NPs can greatly enhance the photo-induced charge carriers in ZnO NPs and thus promoting their ROS generation. Their result demonstrated that constructing hybrid nanostructures with Au is an efficient strategy to improve the photodynamic therapeutic effect of metal oxide. Au-TiO_2_ NPs is another Au-based hybrid nanostructure with certain physicochemical properties outperforming their respective building blocks. For example, Yin *et al.* designed Au-TiO_2_ nanostructures and explored their ROS generation capacity under ultrasound stimulation 166. Their results revealed that the hybrid structures exhibited higher ROS generation efficiency and more significant tumor suppression effect than their counterparts without Au growth, demonstrating the potential of using Au-TiO_2_ nanocomposite as sonosensitizer for cancer therapy. Another interesting Au NPs based-hybrid nanostructure is Au-rGO, as demonstrated by Lim *et al.*, where rGO was coated over Au nanorod 167, 168. Due to high thermal conductivity of rGO and LSPR characteristic of Au nanorods, the prepared Au-rGO hybrid nanostructures exhibited amplified photothermal effect and PA signal intensity, compared to pure Au or graphene oxide/reduced graphene oxide. Moreover, Au can also be integrated with silica layer to form Au@silica nanostructure 169, thus combining the high drug loading capacity of silica with strong photothermal response of Au nanostructures, leading to better cancer cell killing outcome due to the synergistic effect of photothermia and NIR-induced drug release.

## Limitations and challenges

As reviewed briefly, many Au-based nanohybrids with enhanced physicochemical properties and bioactivities have been developed to date, which possess the potential to significantly improve cancer treatment outcomes. However, many challenges need to be resolved before they can be successfully translated to clinical usages.

### Synthetic challenges

Many synthetic issues remain to be addressed before we can explore the nanohybrids unique properties for nanomedicine. Although a rich library of noble metal Au-based nanocomposites is now available, their syntheses are generally complex with many reaction variables to tune. One critical question is how to establish a facile and general synthetic method that can build up the nanohybrids with the right functional building blocks of proper size, interface, and geometry 170. Currently, the typical seeded growth route relies on depositing the second component on the seed NP nucleated *in situ* or synthesized in advance, which can be severely limited by the interfacial energy or lattice matching requirements of the different crystalline domains. Moreover, the growth kinetics can be influenced by complicate synthetic conditions such as reaction temperature, concentration ratio of growth material to seeds NPs, and surface property of the seeds. Non-optimized procedures may lead to low yield of nanohybrids at the end of long tedious procedures. Therefore, developing general synthetic route and establishing standardized protocols to reliably prepare high-quality noble metal-based nanocomposites with controllable morphologies is highly desirable for their extensive biomedical applications.

### Biosafety

For clinical applications, the critical pharmacological behaviors such as biodistribution and biosafety of inorganic nanomaterials remain an under-explored territory. The physicochemical attributes such as NP size, shape, and surface coating are known to affect their cellular uptake, biodistribution, and nanotoxicity. This calls for systematic investigation on the *in vivo* behaviors of designed nanocomposites. The choice of chemical composition and surface coating is clearly critical for the nanocomposites biocompatibility. In terms of composition, Au NPs are generally considered to be bioinert, while copper chalcogenides and metal oxides may be etched or biodegraded in the body fluid, releasing metal ions and introducing potential toxicity to cells and organs. On the other hand, nanotoxicity is also strongly influenced by NP surface modifications. Surface coating can induce cytotoxicity effect directly or indirectly by influencing the formation of protein corona, and the subsequent cellular internalization and final fate of the NPs. As many of the NP physicochemical properties are highly interconnected, it is challenging to evaluate the cytotoxic effect originating from one single attribute of the NPs. Moreover, issues on the long-term metabolism of inorganic nanomaterials such as decomposition, degradation, and clearance of the nanocomposites from the body need to be addressed before they can be applied for clinical usage 171-177. Although many cytotoxicity studies on Au NPs have suggested that they possess good biological safety within several weeks, a great risk of the bioinert NPs is that they may stay in the body and induce chronic toxicity over extended time. Therefore, a balanced stability, slow degradation, and fast clearance should be considered for nanohybrids design with proper choice of chemical composition and surface coating. Finally, the *in vitro*/*in vivo* models employed in the biosafety evaluation can also influence the behavior and fate of the hybrid NPs, which may render conflicting results. To obtain accurate and consistent nanotoxicity evaluation, establishing standardized and reliable protocols to systematically investigate the impact of pharmacological parameters of the NPs is fundamentally important for the biosafety study of the hybrid NPs.

## Conclusions and perspectives

Nanohybrids composed of noble metal Au and copper chalcogenides or magnetic metal oxides have emerged as a unique class of material due to their interesting plasmon-magnetic properties, and the combined diagnostic and therapeutic functional units in one single entity. In this short review, we have summarized some recent developments in building up Au-based inorganic nanohybrids with controlled composition and structure, and highlighted progresses made in their theranostics applications.

Despite substantial progresses that have been made in the field of Au-based hybrid nanomedicine, this field is still at a rather preliminary stage from the standpoint of practical medical applications, especially in terms of biosafety that we have pointed out in the previous section. To address these important questions, several issues need to be addressed. First of all, more comprehensive studies need to be focused on the nanomaterial-biological system interactions, in order to better understand the critical factors determining the biosafety of nanocomposites, which will then be used for better nanomedicine design. Although plenty nanotoxicity studies have already been carried out on Au 36, 178-181, whether the attachment of a second component would affect its cellular behavior and final fate within organisms is still uncertain. It is believed that many physicochemical parameters of nanomaterials such as size, shape, charge, and surface modification can greatly influence the biocompatibility of nanocomposites. Future toxicity investigations on noble metal Au-based nanocomposites should consider all of these complex factors and explore the underlying molecular mechanisms of various factors on gene expression, signaling pathways, and downstream cell metabolism. In addition, when interpreting the interaction between nanocomposites and organism, it is necessary to note that the organisms may behave well and show normal physiological functions for a short period 182. However, the organisms may suffer subtle but irreversible changes in their genetics after continuous exposure. Therefore, additional long-term toxicity evaluation is needed in the corresponding animal experiments. Moreover, excellent bioavailability and targeting ability is of great importance for highly efficient biomedical nanotechnologies. The hybrid nanocomposites must avoid rapid clearance during blood circulation and increase their accumulation dosage at the desirable target site. Recently, the cell membrane-cloaking strategy by mimicking nanoparticles with erythrocyte or host cancer cell membrane envelopes has shown great potency in increasing circulation time by inhibiting macrophage recognition and improving targeting ability via homotypic binding 183-187. However, this technique still faces some inherent challenges. For example, the detailed biomolecular mechanism of the homotypic binding derived from cell membrane is yet unclear. Identification of the specific ligands involved in the host membrane recognition would benefit future development of nanomaterial-based biomimetic nanotechnology. Furthermore, it is very difficult for the nanohybrids to go deep into solid tumors, which severely limits their efficacy as drug carrier and imaging platform. Knowledge on the NPs' pathway into tumors would be useful in helping design nanohybrids structures with improved tumor penetration depth. Despite the discovery of endothelial gaps in tumor vasculatures using developed animal models, nanomedicine design utilizing the enhanced permeability and retention (EPR) effect for human tumor treatment has been controversial. Only a few anticancer nanomedicines have received approval for clinical application based on EPR effect. Recently, new evidence has emerged, suggesting NPs may enter tumors via an active process through endothelial transcytosis 188. These observations may establish new paradigms and enable novel strategies to help expedite the clinical translation of nanomedicine.

Au-based multifunctional nanocomposites have shown their promises in both early diagnostic and theranostic applications. Of note, their manifested multifunctionality due to the synergistic effect between different components would enable safer and more effective theranostic treatment. Undoubtedly, with continuing endeavor in the design and development of new multifunctional nanohybrids, it is our firm belief that they hold great diagnostic and therapeutic potential in broad biomedical applications, and are likely to find real significance in the new era of personalized precision nanomedicine.

## Figures and Tables

**Table 1:** Summary of Au-based inorganic hybrid nanostructures used in nanomedicine

Hybrid structure	Synthetic method	Size	Application	Advantage	Ref.
Au-Cu_9_S_5_ UFO-shape	Seeded growth method	~22 nm (TEM)	PTT-CT	Improved heating effect	77
Au-Cu_2-x_Se heterodimer	Seeded growth method	~10 nm (TEM)	PA	Deep tissue imaging up to 17 mm	78
Au-Cu_2-x_S core-shell	Anion exchange	85.87±10 nm (DLS)	SERS/PA-PTT	Bimodal imaging-guided PTT	73
Au-Cu_9_S_5_@MSN	Seeded growth method	107 nm (DLS)	PTT-MRI	MRI tacking drug release	80
CuS@Cu_2_S@Au hollow structure	Template method	100 nm (TEM)	PTT-chemotherapy	Photo-switchable targeting	96
Au-Cu_2-x_S/Se core-shell or heterodimer	Seeded growth method	Tunable (TEM)	N.A	N.A	102
Au@Cu_2-x_S/Se core-shell	Self-assembly	Tunable (TEM)	PTT-PA-CT	High photothermal conversion efficiency	103
Cubic CuS@spicky Au core-shell	Template method	78±5 nm (DLS)	PTT-SERS	Enhanced PTT and SERS	109
Au-CuS yolk-shell structure	Template method and anion exchange	Tunable (TEM)	PTT-PDT-chemotherapy	Enhanced PTT and PDT	110
Au-Cu_2-x_S core-shell	Template method	150 nm (TEM)	Chemo-PTT	Enhanced photothermal effect	111
Fe_3_O_4_@Au core-shell	Seeded growth method	6.3 ± 0.7 nm (TEM)	Hyperthermia	Improved hyperthermia	121
Fe_3_O_4_/Au cluster/shell	Seeded growth method	240 nm (TEM)	SERS-magnetic hyperthermia	Improved hyperthermia	122
Fe@Au bi-layer semi-shell	Nanolithography and physical vapor deposition	40 nm (TEM)	CT, MRI, and fluorescence	Magnetically amplified photothermal therapy	123
Fe_3_O_4_/Au cluster/shell	Seeded growth method	126±11 nm (TEM)	PTT-magnetic hyperthermia	Bimodal thermo-therapy	124
Au-Fe_3_O_4_ heterodimer	Seeded growth method	11-14 nm (TEM)	MRI-CT	Bimodal imaging	136
Fe_3_O_4_@Au core-shell	One-pot hydrothermal	262.7±3.06 nm (DLS)	MRI-CT	Bimodal imaging	137
Fe_2_O_3_@Au core-shell	Seeded growth method	22.1±1.9 nm (TEM)	MRI-CT	Bimodal imaging	138
Fe_3_O_4_@SiO_2_@Au core-shell	Seeded growth method	222±1.5 nm (DLS)	MRI/CT(PA) imaging	Bimodal imaging	140
Fe_2_O_3_@Au core-shell	Seeded growth method	179 nm (DLS)	SERS-PA-MRI-PTT	Tri-modal imaging-guided PTT	141
Fe_3_O_4_@Au yolk-shell	Seeded growth method	65 nm (TEM)	MRI-PA-PET-chemo-thermal therapy	Multimodal imaging-guided chemo-thermal therapy	142
Fe_3_O_4_@Au@mSiO_2_ core-shell	Seeded growth method	10.4 ± 2.3 nm (DLS)	PTT-PDT	Enhanced PDT	143
Au-Fe_3_O_4_ heterodimer	Seeded growth method	16.7 ± 2.3 nm (TEM)	X-ray protection and X-ray enhancing agents	Discriminate healthy cell and cancer cell	144
MSN-Au-Fe_3_O_4_ core-shell	Assembly	140 nm (TEM)	Nanozyme	Nanozyme-catalyzed cascade reaction	145
Au@MnO_2_ core-shell	Seeded growth method	50 nm (TEM)	Radiotherapy	Overcoming the hypoxia-associated radiotherapy resistance	158
Au cage@MnO_2_ core-shell	Seeded growth method	91 nm (TEM)	PDT	Boost immunogenic PDT	159
Cu_2-x_Se (Au)@MnO_2_ core-shell	Seeded growth method	60 nm (TEM)	PTT	Redox-activated MRI-guided PTT	162
Au@MnO_2_ UFO-shaped	Seeded growth method	230 nm (TEM)	Dark field imaging	Monitoring cell membrane vesiculation	163
Au@MnO_2_ core-shell	Bio-templated method	20-25 nm (TEM)	Fluorometric and MRI based sensing	Inherent cross-validation	164

Abbreviations: PTT: photothermal therapy; PDT: photodynamic therapy; PA: photoacoustic imaging; CT: computed tomography; MRI: magnetic resonance imaging; SERS: surface enhanced Raman scattering; MSN: mesoporous silica nanoparticle; TEM: transmission electron microscopy; DLS: dynamic light scattering.
